# Kentucky Lunatic Asylum

**Published:** 1844-01

**Authors:** 


### KENTUCKY LUNATIC ASYLUM.

A brighter day seems to have dawned upon this institution. The Annual Report of the Managers and attending Physicians, for 1843, exhibits an improvement in its administration which will fill many a heart in this Commonwealth with joy. The humane and enlightened practice long since adopted in other retreats for the insane appears, at length, to have found its way into the sylum at Lexington, and is attended with the same happy results—the restoration of many of its unfortunate inmates to reason. It was not so in former times. If one recovered, it seemed to be by accident, or, at the most, by the *vis medicatrix naturæ.* Nor could it well be otherwise. No adequate medical attendance was provided. The salary allowed to the physician who attended was so small that he could not afford to bestow much of his time upon the institution, and no systematic plan was adopted and persevered in for the cure of the insane. In these years—the long period from 1824, when the charity was opened, to 1843, when the present attending physicians entered upon their duties—according to to the report before us, the deaths in the asylum, annually, exceeded 39 per cent. During the past year, the deaths have amounted to a small fraction over 4 per cent. This, as the attending physicians justly observe, is "*a remarkable difference.*" There must be a cause for it, and is doubtless to be found in the circumstances stated in the report.

First, the attending physicians, Drs. Pinckard, Letcher, and Price, on assuming the care of the institution, set about improving the general health of the patients. In the second place, they sought to give the inmates of the house all the opportunities for exercise and amusement which the institution afforded. They found that the patients were addicted to the intemperate use of tobacco, and that in nearly every one the general health was seriously impaired by the indulgence. This practice was interdicted, and, the report informs us, the result was soon seen in the comparatively ruddy and robust appearance of those who a short time before were pale and emaciated. Many of the lunatics, too feeble to walk, were evidently suffering for the want of exercise, and from confinement, "for years, in the narrow cells of the asylum." On application by the physicians to the commissioners of the Hospital, carriages were provided for this class of patients. Again, continues the report of the physicians, "as we ourselves are fond of good living, we directed the patients generally to be fed on the best beef and mutton the market affords, and they are now seated at as fine a table as any hotel in Kentucky can furnish." The remedial treatment has been applied with great punctuality and discrimination, and with results, as contrasted with the success of previous years, to which the present faculty of the asylum may appeal with a feeling of triumph. The moral treatment has also undergone a beneficial reform. As an indispensable preliminary to the introduction of the best system, the physicians have commenced a classification of the patients, and although this is not yet perfected, they express a belief that the measure is so far matured, that the best effects may be expected to be developed by it in the future operations of the establishment. At the present time, constant and agreeable occupation is provided for the insane; those who are too feeble to perform manual labor are taken daily in carriages, or other vehicles, into the open air; music and dancing parties have been occasionally introduced with advantage; chains, stripes, and box-houses have been abolished, and, in their stead, a plan of government has been adopted, styled, in the report, "patriarchal"—one characterized by all the respect and kindness towards the afflicted compatible with firmness and decision. Such is the cheering picture given in the report, of the present state and future promise of the Kentucky Lunatic Asylum. We add a few details.

The number of patients in the charity at the beginning of 1843 was 156, to which number 74 were added subsequently, making 230 for the whole year—a larger number, by 20 per cent., than was ever before in the asylum. Of these, 15 died, 33 were cured, 16 are reported as improved in their condition, and 6 were taken home. Of the deaths, 3 occurred in January, before the present attending physicians assumed their duties in the institution, and 4 occurred so soon afterwards, that they insist on not being held responsible for them. Deducting these from the whole number, it leaves but 8 for the eleven months of their attendance. Of the patients in the house at the beginning of 1843, and admitted during the year, the insanity of 154 was of long standing—that of 34, recent. Among the old cases, 8 per cent. were restored, 2 per cent. were improved, and 5 deaths occurred. The recent cases afforded of cures over 58 per cent., of improvement 6 per cent., and of deaths nearly 3 per cent.—fractions being omitted in every instance. The number in the asylum at the end of the year, was 170; of whom 100 are males, and 70 females. The commissioners speak of a farm, rented last year, as having been of incalculable benefit to the patients, affording them the means of healthful labor, and more ample scope for exercise, and they conclude by suggesting that further improvements might still be made in the economy of the asylum, and by recommending to the Legislature, in strong terms, the purchase of fifty or sixty additional acres of land for the use of the institution.

We have been thus particular in our account of this important State institution, because it is an object of deep public interest. We are very often consulted by our friends in other States, as to the most eligible retreat for insane persons, and it affords us unmixed pleasure to be able to present them with so favorable a description of the Asylum at Lexington. In the neighborhood of one of the most beautiful towns in the country, and in the bosom of a cultivated, refined and moral community, with all the advantages of an airy and salubrious situation, there is no reason why, with the proper interior regulations, such as seem to have been adopted, it should not enjoy as much success in restoring the insane to their sound minds as any asylum in the land. Y.

